# Association of joint trajectories of dietary diversity and physical activity with cognitive function in older adults: a prospective cohort study

**DOI:** 10.3389/fpubh.2026.1800751

**Published:** 2026-04-16

**Authors:** Chuang Shen, Yifan Yin, Song Lin

**Affiliations:** 1 School of Physical Education, Chongqing University of Posts and Telecommunications, Chongqing, China; 2 School of Art and Design, Chongqing College of International Business and Economics, Chongqing, China; 3 Office of Science and Technology, Chongqing University of Posts and Telecommunications, Chongqing, China

**Keywords:** cognitive function, dietary diversity, older adults, physical activity, prospective cohort study, trajectory analysis

## Abstract

**Background:**

Cognitive impairment represents a major public health challenge affecting older adults' health and quality of life. Dietary diversity and physical activity are important modifiable lifestyle factors; however, previous studies have pre-dominantly relied on single time-point assessments, neglecting longitudinal behavioral changes. This study investigated the association between joint longitudinal trajectory patterns of dietary diversity and physical activity and cognitive function in older adults.

**Methods:**

Using data from four waves (2008–2018) of the Chinese Longitudinal Healthy Longevity Survey, we included 1,826 older adults aged ≥65 years with normal baseline cognitive function. Latent class growth analysis identified distinct trajectory patterns of dietary diversity score (DDS) and physical activity (PA) from 2008 to 2014, which were cross-classified into joint trajectory groups. Cognitive impairment (education-stratified MMSE cutoffs) and continuous MMSE scores in 2018 served as outcomes. Modified Poisson regression and multivariable linear regression examined associations between joint trajectories and cognitive function.

**Results:**

Two DDS trajectories (high-stable: 53.5%; low-stable: 46.5%) and two PA trajectories (high: 37.2%; low: 62.8%) formed four joint trajectory groups. Over 10-year follow-up, cognitive impairment incidence was 18.1%. Compared with the “High DDS and High PA” reference group, the “High DDS and Low PA” group (*RR* = 1.48, 95% CI: 1.10–2.01) and “Low DDS and Low PA” group (*RR* = 1.41, 95% CI: 1.03–1.93) showed significantly elevated cognitive impairment risks. For continuous MMSE scores, the “High DDS and Low PA” group (β = −1.12, 95% CI:−1.94 to −0.30) and “Low DDS and Low PA” group (β = −1.20, 95% CI: −2.04 to −0.36) demonstrated significantly poorer cognitive function. Sensitivity analyses confirmed these findings.

**Conclusions:**

Maintaining high dietary diversity with regular physical activity was associated with optimal cognitive function. Physical inactivity emerged as an independent risk factor for cognitive decline that favorable dietary habits could not fully offset. These findings support integrated cognitive health strategies combining dietary intervention with physical activity promotion.

## 1 Introduction

With the accelerating global population aging, cognitive impairment and its end-stage manifestation—dementia—have emerged as one of the most formidable public health challenges of the 21st century. According to the latest estimates from the World Health Organization, approximately 55 million individuals worldwide are living with dementia, with projections indicating an increase to 78 million by 2030 and 158 million by 2050, approximately 60% of whom reside in low- and middle-income countries (1). China has the world's largest older adults population, with over 200 million individuals aged 65 years and above, accounting for approximately 14.9% of the total population. Among this population, the prevalence of mild cognitive impairment is 15.5% and dementia prevalence is 6.0%, imposing a substantial burden on family caregiving and healthcare systems (2). The 2024 Lancet Commission on Dementia Prevention, Intervention, and Care reported that approximately 45% of dementia cases are attributable to 14 potentially modifiable risk factors (3). This finding provides an important public health opportunity for primary prevention of dementia and underscores the significance of lifestyle interventions across the life course.

Dietary diversity is a comprehensive indicator of diet quality, reflecting the variety and balance of different food types consumed by an individual over a specific period (4). A diversified diet provides abundant antioxidant nutrients, and the WHO recommends maintaining dietary diversity to prevent malnutrition and chronic diseases (5). Prospective studies have demonstrated that higher dietary diversity is significantly associated with better cognitive function (6, 7). Systematic reviews and meta-analyses have established that greater dietary diversity is significantly associated with improved cognitive function and reduced dementia risk (8). Physical activity represents another critical modifiable lifestyle factor. Regular physical exercise exerts neuroprotective effects through multiple biological mechanisms: enhancing cerebral blood flow and oxygen supply, promoting secretion of brain-derived neurotrophic factor (BDNF) and insulin-like growth factor-1 (IGF-1), stimulating hippocampal neurogenesis and synaptic plasticity, improving insulin sensitivity and glucose metabolism, reducing chronic low-grade inflammation, and facilitating β-amyloid clearance (9–12). Pooled analyses of prospective cohort studies have shown that compared with the lowest level of physical activity, the highest level is associated with an approximately 28% reduction in all-cause dementia risk (13).

Notably, health behaviors exhibit marked clustering patterns. Individuals who engage in one health behavior are more likely to simultaneously practice other healthy behaviors, resulting in synergistic or antagonistic health effects (14). Previous studies have pre-dominantly employed composite indices such as “healthy lifestyle scores,” which assume that the effects of individual behaviors are linearly additive, potentially obscuring heterogeneity among different behavioral combination patterns (15, 16). Furthermore, conventional studies have largely relied on single time-point exposure assessments, disregarding the dynamic characteristics of lifestyle behaviors over time (16). Dietary habits and physical activity capacity among older adults may undergo significant changes with advancing age, alterations in health status, and modifications in living environment, rendering static exposure measurements inadequate for capturing these longitudinal evolutionary patterns.

The data analyzed in this study were derived from the Chinese Longitudinal Healthy Longevity Survey (CLHLS), a nationwide longitudinal study jointly conducted by the Institute of Healthy Aging and Development and the National School of Development at Peking University. The CLHLS collects extensive information on the demographic characteristics, health status, and lifestyle behaviors of older adults across China. Using the CLHLS dataset, the present study is among the first to apply Latent Class Growth Analysis (LCGA) to simultaneously identify longitudinal trajectory patterns of dietary diversity and physical activity. Based on these trajectories, joint trajectory groups were constructed to examine how different diet–exercise behavioral combinations are associated with the risk of cognitive impairment and levels of cognitive function in older adults. By capturing the dynamic evolution of lifestyle behaviors over time, this study provides new evidence to support the development of precision-targeted and integrated strategies for promoting cognitive health in aging populations.

## 2 Methods

### 2.1 Study population

**Figure 1:**
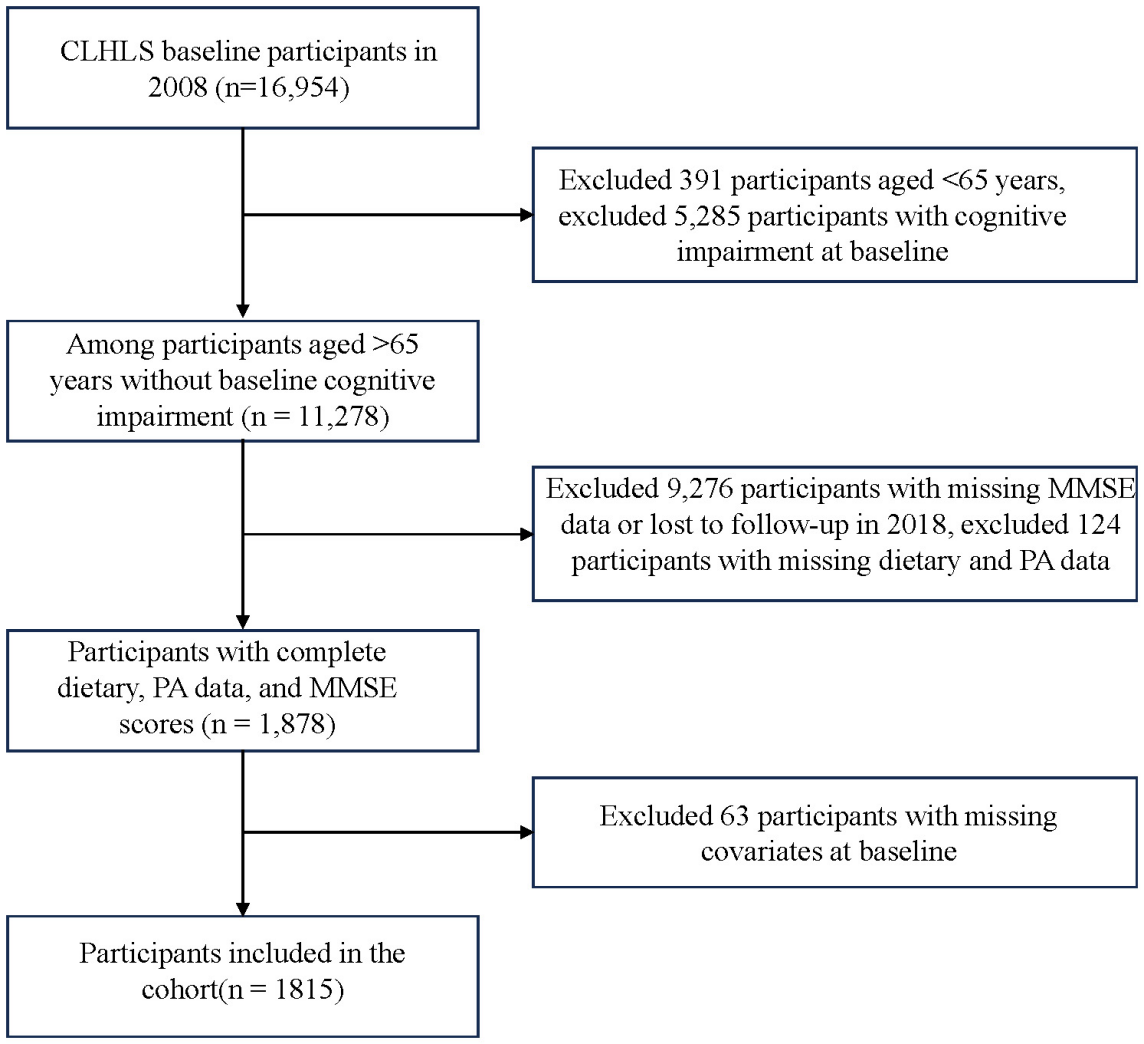
Flowchart of sample selection procedure.

Data for this study were derived from the CLHLS, conducted across 23 provinces, municipalities, and autonomous regions in China. Initiated in 1998, the CLHLS employs a multi-stage stratified random sampling methodology to survey individuals aged 65 years and above through in-home interviews. The survey encompasses multiple dimensions including demographic characteristics, socioeconomic status, lifestyle factors, dietary habits, functional status, cognitive function, mental health, and chronic disease history, with data collected by uniformly trained interviewers via face-to-face interviews (17). This study utilized data from four survey waves: 2008 (baseline), 2011, 2014, and 2018. Participants meeting the following criteria were included: (1) aged ≥65 years at baseline; (2) normal baseline cognitive function (defined using education-stratified MMSE cutoffs: illiterate ≥18, primary school ≥21, secondary school and above ≥24); (3) complete dietary frequency and physical activity data across the 2008, 2011, and 2014 waves; (4) no deaths or participant dropouts occurred during the follow-up period; and (5) complete cognitive function assessment data in 2018. Ultimately, 1,826 participants were enrolled in this study, with the flowchart presented in Figure 1.

### 2.2 Dietary diversity

The Dietary Diversity Score (DDS) was employed to assess dietary diversity among older adults, calculated based on consumption frequency of nine food categories from the CLHLS questionnaire: fresh fruits, vegetables, meat, fish, eggs, legume products, tea, garlic, and salted vegetables. The scoring criteria were as follows: for the first eight healthy food categories, a consumption frequency of “daily” or “almost daily” was scored as 1 point; salted vegetables employed reverse scoring, with “rarely” or “never” consumption scored as 1 point. Total DDS ranged from 0 to 9 points, with higher scores indicating better dietary diversity (18). This scoring methodology has demonstrated good content validity and test-retest reliability in Chinese older adult populations and has been validated in multiple studies (19).

### 2.3 Physical activity

Physical activity (PA) was assessed using the CLHLS questionnaire item “Do you currently engage in physical exercise?” Responses of “yes” were coded as 1 (regular exercise), while responses of “no” were coded as 0 (no regular exercise). Although this indicator is relatively simplified, it demonstrates acceptable operability and epidemiological validity in large-scale epidemiological surveys (20).

### 2.4 Outcome assessment

Cognitive impairment was determined using education-adjusted Chinese Mini-Mental State Examination (CMMSE) scores. Participants were assessed by trained and certified staff using the CMMSE, which comprises 24 items covering cognitive domains including temporal orientation (5 points), spatial orientation (5 points), immediate recall (3 points), attention and calculation (5 points), delayed recall (3 points), language ability (8 points), and visuospatial ability (1 point), with a total score ranging from 0 to 30. Given the substantial influence of educational attainment on MMSE performance, education-stratified diagnostic cutoffs were employed: illiterate (0 years of education) MMSE < 18, primary school (1–6 years) MMSE < 21, and secondary school and above (>6 years) MMSE < 25 were defined as cognitive impairment (21, 22).

### 2.5 Covariates

Based on prior literature, the following potential confounders were included as covariates: (1) demographic characteristics: age, sex (male/female), educational attainment (illiterate/primary school/secondary school and above), marital status (currently married/not currently married), and residence (urban/town/rural); (2) lifestyle factors: smoking status (yes/no) and alcohol consumption status (yes/no); (3) nutritional status: body mass index (BMI, categorized as underweight < 18.5, normal weight 18.5–23.9, and overweight/obese ≥24 kg/m^2^); and (4) chronic diseases: hypertension (yes/no), diabetes mellitus (yes/no), heart disease (yes/no), and stroke (yes/no).

### 2.6 Statistical analysis

Baseline characteristics were summarized as means ± standard deviations for continuous variables and frequencies (percentages) for categorical variables. Latent class growth analysis (LCGA) was performed separately on longitudinal DDS and PA data (2008–2014) to identify distinct trajectory groups, assuming individuals within the same class follow identical growth trajectories with within-class variance fixed at zero. Model selection integrated multiple fit indices (AIC, BIC, aBIC; lower values preferred), entropy (>0.70), average posterior probabilities (>0.70), and minimum class size (≥5%), balanced with theoretical interpretability. Two DDS trajectories (high-stable, low-stable) and two PA trajectories (high, low) were identified and cross-classified to create four joint trajectory groups: “High DDS and High PA” (reference), “High DDS and Low PA”, “Low DDS and High PA”, and “Low DDS and Low PA”. Although the entropy value of the DDS trajectory model (0.53) was relatively low, the two-class solution was selected due to its good interpretability and satisfactory classification quality, with average posterior probabilities greater than 0.85.

Modified Poisson regression with robust variance estimation was employed to estimate relative risks (*RR*) and 95% confidence intervals (CI) for cognitive impairment across trajectory groups. Multivariable linear regression examined associations with continuous MMSE scores. Three sequential models were constructed: model 1 (unadjusted), model 2 (adjusted for demographic variables), and Model 3 (additionally adjusted for lifestyle factors and chronic disease multimorbidity). Sensitivity analyses included: (1) application of a unified MMSE cutoff (< 24); (2) application of a stricter cutoff (< 18); (3) exclusion of participants with baseline MMSE < 24; and (4) exclusion of participants with baseline MMSE ≥28.

All analyses were conducted using R version 4.3.0 (lcmm and sandwich packages). Two-sided *P* < 0.05 was considered statistically significant.

## 3 Results

### 3.1 Baseline characteristics of the study population

Among the 1,826 participants, the mean baseline age was 74.76 ± 7.54 years, and 52.4% (*n* = 956) were female. Significant differences in baseline characteristics were observed across joint trajectory groups (Table 1). Participants in the “High DDS and High PA” group were younger, had a higher proportion of males (59.1%), higher educational attainment (secondary school and above: 29.0%), higher proportion of urban residents (33.4%), higher proportion of currently married individuals (67.7%), and higher prevalence of overweight/obesity (30.3%). Conversely, the “Low DDS and Low PA” group had the highest proportion of females (61.7%), illiterate individuals (61.1%), rural residents (83.4%), and underweight individuals (30.7%), along with the lowest baseline DDS and PA levels.

**Table 1:** Baseline characteristics of participants by joint trajectory groups.

Characteristic	Overall (*N* = 1,826)	High DDS and High PA (*n* = 452)	High DDS and Low PA (*n* = 524)	Low DDS and High PA (*n* = 228)	Low DDS and Low PA (*n* = 622)	*p*
Sample size, *n*	1,826	452	524	228	622	
Age [mean (SD)]	74.76 (7.54)	74.26 (7.32)	74.63 (7.58)	74.99 (7.43)	75.16 (7.69)	0.252
Sex (%)						
Male	870 (47.6)	267 (59.1)	266 (50.8)	99 (43.4)	238 (38.3)	< 0.001
Female	956 (52.4)	185 (40.9)	258 (49.2)	129 (56.6)	384 (61.7)	
Education level (%)						
Illiterate	860 (47.1)	131 (29.0)	234 (44.7)	115 (50.4)	380 (61.1)	< 0.001
Primary school	718 (39.3)	190 (42.0)	223 (42.6)	96 (42.1)	209 (33.6)	
Middle school or above	248 (13.6)	131 (29.0)	67 (12.8)	17 (7.5)	33 (5.3)	
Marital status (%)						
Without spouse	759 (41.6)	146 (32.3)	193 (36.8)	119 (52.2)	301 (48.4)	< 0.001
With spouse	1,067 (58.4)	306 (67.7)	331 (63.2)	109 (47.8)	321 (51.6)	
Residence (%)						
Urban	239 (13.1)	151 (33.4)	39 (7.4)	33 (14.5)	16 (2.6)	< 0.001
Town	363 (19.9)	119 (26.3)	105 (20.0)	52 (22.8)	87 (14.0)	
Rural	1,224 (67.0)	182 (40.3)	380 (72.5)	143 (62.7)	519 (83.4)	
BMI category (%)						
Overweight/obese	387 (21.2)	137 (30.3)	108 (20.6)	49 (21.5)	93 (15.0)	< 0.001
Underweight	380 (20.8)	59 (13.1)	95 (18.1)	35 (15.4)	191 (30.7)	
Normal	1,059 (58.0)	256 (56.6)	321 (61.3)	144 (63.2)	338 (54.3)	
Smoking (%)						
Non-smoker	1,419 (77.7)	345 (76.3)	396 (75.6)	178 (78.1)	500 (80.4)	0.215
Smoker	407 (22.3)	107 (23.7)	128 (24.4)	50 (21.9)	122 (19.6)	
Drinking (%)						
Non-drinker	1,411 (77.3)	325 (71.9)	403 (76.9)	179 (78.5)	504 (81.0)	0.005
Drinker	415 (22.7)	127 (28.1)	121 (23.1)	49 (21.5)	118 (19.0)	
Hypertension (%)						
No	1,412 (77.3)	332 (73.5)	411 (78.4)	168 (73.7)	501 (80.5)	0.022
Yes	414 (22.7)	120 (26.5)	113 (21.6)	60 (26.3)	121 (19.5)	
Diabetes (%)						
No	1,780 (97.5)	431 (95.4)	512 (97.7)	222 (97.4)	615 (98.9)	0.004
Yes	46 (2.5)	21 (4.6)	12 (2.3)	6 (2.6)	7 (1.1)	
Heart disease (%)						
No	1,658 (90.8)	394 (87.2)	488 (93.1)	196 (86.0)	580 (93.2)	< 0.001
Yes	168 (9.2)	58 (12.8)	36 (6.9)	32 (14.0)	42 (6.8)	
Stroke (%)						
No	1,747 (95.7)	433 (95.8)	506 (96.6)	208 (91.2)	600 (96.5)	0.005
Yes	79 (4.3)	19 (4.2)	18 (3.4)	20 (8.8)	22 (3.5)	
DDS score [mean (SD)]	5.17 (1.85)	6.41 (1.46)	6.02 (1.43)	4.13 (1.41)	3.93 (1.56)	< 0.001
Regular PA [mean (SD)]	0.36 (0.48)	0.75 (0.44)	0.15 (0.35)	0.66 (0.48)	0.14 (0.35)	< 0.001

### 3.2 Dietary diversity score trajectories

**Figure 2:**
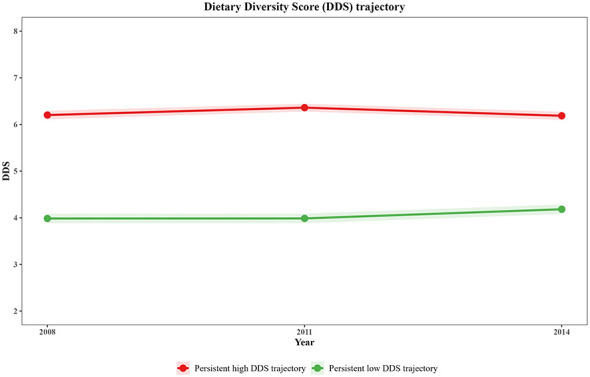
Dietary diversity score trajectories of older people.

As shown in Supplementary Table S1, LCGA model fit results indicated that the two-class model demonstrated optimal BIC (21,678.25). Although entropy (0.53) was slightly below the ideal criterion of 0.7, average posterior probabilities for each class exceeded 0.85, and the proportion of individuals with high probability (>0.7) exceeded 79%, indicating acceptable classification accuracy. The DDS trajectories are illustrated in Figure 2. Two distinct DDS trajectory groups were identified: (1) “High DDS stable trajectory” group (*n* = 976, 53.5%): baseline mean DDS of 6.20 points, remaining stable throughout follow-up, representing a population that consistently maintained high dietary diversity; and (2) “Low DDS stable trajectory” group (*n* = 850, 46.5%): baseline mean DDS of 3.98 points, showing slight improvement over follow-up but consistently remaining below the high DDS group.

### 3.3 Physical activity trajectories

**Figure 3:**
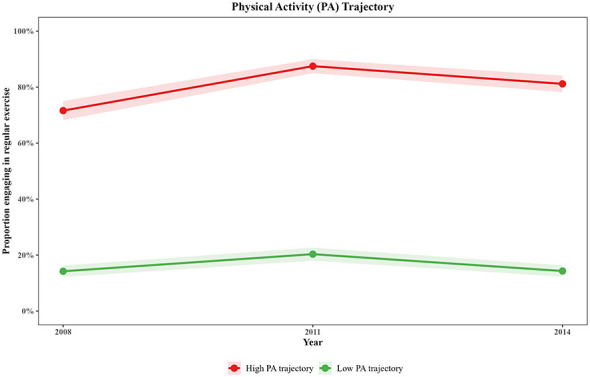
Physical activity trajectories of older people.

As shown in Supplementary Table S2, LCGA model fit results similarly supported the two-class model for PA trajectories (BIC = 7,156.11, entropy = 0.687). The PA trajectories are depicted in Figure 3. Two distinct PA trajectory groups were identified: (1) “High PA trajectory” group (*n* = 680, 37.2%): baseline regular exercise rate of 71.6%, maintaining high levels throughout follow-up; and (2) “Low PA trajectory” group (*n* = 1,146, 62.8%): baseline regular exercise rate of 14.2%, consistently remaining at low levels throughout follow-up.

### 3.4 Distribution of joint trajectory groups

Cross-classification of DDS and PA trajectories yielded four joint trajectory groups: “High DDS and High PA” (*n* = 452, 24.8%), “High DDS and Low PA” (*n* = 524, 28.7%), “Low DDS and High PA” (*n* = 228, 12.5%), and “Low DDS and Low PA” (*n* = 622, 34.1%). Notably, more than one-third of older adults simultaneously exhibited low dietary diversity and lacked regular exercise habits, representing a dual unfavorable lifestyle combination.

### 3.5 Association between joint trajectory groups and cognitive impairment risk

Over the 10-year follow-up period, 331 participants developed cognitive impairment, yielding an overall incidence of 18.1%. Cognitive impairment incidence exhibited a clear gradient across joint trajectory groups: the “High DDS and High PA” group had the lowest incidence (12.6%, 57/452), followed by the “Low DDS and High PA” group (18.9%, 43/228) and the “High DDS and Low PA” group (19.7%, 103/524), with the “Low DDS and Low PA” group demonstrating the highest incidence (20.6%, 128/622).

Results from modified Poisson regression analyses are presented in Table 2. Compared with the “High DDS and High PA” reference group, the main association patterns remained stable in the fully adjusted model after additional adjustment for lifestyle factors and multimorbidity: the “High DDS and Low PA” group maintained a significantly elevated cognitive impairment risk of 48% (*RR* = 1.48, 95% CI: 1.10–2.01, *P* = 0.009); the “Low DDS and Low PA” group demonstrated a 41% increased risk (*RR* = 1.41, 95% CI: 1.03–1.93, *P* = 0.019); the “Low DDS and High PA” group showed a 33% increased risk that did not reach statistical significance (*RR* = 1.33, 95% CI: 0.92–1.93, *P* = 0.079).

**Table 2:** Association between joint trajectory groups and risk of cognitive impairment.

Joint trajectory groups	Events/participants	Model 1	*p*	Model 2	*p*	Model 3	*p*
High DDS and high PA	57/452	1.00 (Reference)		1.00 (Reference)		1.00 (Reference)	
High DDS and low PA	103/524	1.56 (1.16–2.10)	0.004	1.48 (1.09–2.00)	0.012	1.48 (1.10–2.01)	0.009
Low DDS and high PA	43/228	1.50 (1.14–2.15)	0.030	1.37 (0.95–1.98)	0.090	1.33 (0.92–1.93)	0.079
Low DDS and low PA	128/622	1.63 (1.22–2.18)	0.001	1.42 (1.04–1.95)	0.028	1.41 (1.03–1.93)	0.019

Model 1 was unadjusted.

Model 2 adjusted for age, sex, education, marital status, and residence.

Model 3 additionally adjusted for smoking, alcohol consumption, BMI, hypertension, diabetes, heart disease, and stroke based on model 2.

### 3.6 Association between joint trajectory groups and continuous MMSE scores

Multivariable linear regression analyses further corroborated the above findings (Table 3). Using the “High DDS and High PA” group as the reference, the fully adjusted model revealed: the “High DDS and Low PA” group exhibited significantly lower MMSE scores by 1.12 points (β = −1.12, 95% CI: −1.94 to −0.30, *P* = 0.008); the “Low DDS and Low PA” group demonstrated significantly lower MMSE scores by 1.20 points (β = −1.20, 95% CI: −2.04 to −0.36, *P* = 0.005); the “Low DDS and High PA” group showed a trend toward lower MMSE scores that did not reach statistical significance (β = −0.51, 95% CI: −1.55 to 0.52, *P* = 0.330). Notably, in the unadjusted model, the association for the “Low DDS and High PA” group was statistically significant (β = −1.36, *P* = 0.019), but was substantially attenuated following adjustment for demographic variables, suggesting this association may be partially explained by confounding attributable to demographic characteristics.

**Table 3:** Multiple linear regression analysis of MMSE scores across joint trajectory groups.

Joint trajectory groups	Model 1	*p*	Model 2	*p*	Model 3	*p*
High DDS and high PA	1.00 (Reference)		1.00 (Reference)		1.00 (Reference)	
High DDS and low PA	−1.53 (−2.37, −0.70)	< 0.001	−1.08 (−1.90, −0.26)	0.010	−1.12 (−1.94, −0.30)	0.008
Low DDS and high PA	−1.36 (−2.40, −0.32)	0.19	−0.51 (−1.53, 0.51)	0.330	−0.51 (−1.55, 0.52)	0.330
Low DDS and low PA	−2.20 (−2.98, −1.41)	< 0.001	−1.12 (−1.95, −0.29)	0.008	−1.20 (−2.04, −0.36)	0.005

Model 1 was unadjusted.

Model 2 adjusted for age, sex, education, marital status, and residence.

Model 3 additionally adjusted for smoking, alcohol consumption, BMI, hypertension, diabetes, heart disease, and stroke based on model 2.

### 3.7 Sensitivity analyses

Sensitivity analyses generally supported the robustness of the primary findings (Supplementary Table S3). (1) When employing a unified cutoff (MMSE < 24) to define cognitive impairment, the direction of associations remained consistent, though effect sizes were attenuated and did not reach statistical significance, potentially attributable to the substantially elevated cognitive impairment incidence (33.1%) under this definition and increased outcome event heterogeneity. (2) When applying a more stringent cutoff (MMSE < 18), the association pattern was consistent with the primary analysis: both the “High DDS and Low PA” group (*RR* = 1.53, 95% CI: 1.08–2.17, *P* = 0.017) and the “Low DDS and Low PA” group (*RR* = 1.48, 95% CI: 1.04–2.11, *P* = 0.030) demonstrated significantly elevated risks, supporting the robustness of associations in more severe cognitive impairment. (3) Following exclusion of participants with baseline MMSE < 24 (*n* = 1,585), associations were strengthened, and the “Low DDS and High PA” group also achieved statistical significance (*RR* = 1.66, 95% CI: 1.10–2.50, *P* = 0.016), suggesting that the protective effect of physical activity may be more pronounced among populations with better baseline cognitive function.

Sensitivity analyses for continuous MMSE scores were similarly robust (Supplementary Table S4). Following exclusion of participants with baseline MMSE < 24, associations for the “High DDS and Low PA” group (β = −1.07, 95% CI: −1.89 to −0.24, *P* = 0.011) and the “Low DDS and Low PA” group (β = −0.86, 95% CI: −1.69 to −0.03, *P* = 0.042) remained significant. Following exclusion of participants with baseline MMSE ≥28 to mitigate ceiling effects (*n* = 1,278), effect sizes were amplified, with associations for both the “High DDS and Low PA” group (β = −1.55, 95% CI: −2.64 to −0.47, *P* = 0.005) and the “Low DDS and Low PA” group (β = −1.76, 95% CI: −2.81 to −0.71, *P* = 0.001) becoming more pronounced.

## 4 Discussion

Based on 10-year prospective cohort data from the CLHLS, this study is the first to employ latent class growth analysis to simultaneously identify longitudinal trajectory patterns of dietary diversity and physical activity, systematically evaluating associations between different diet-exercise joint trajectories and cognitive function in older adults. The principal findings include: (1) identification of four joint trajectory groups with distinct behavioral characteristics, revealing substantial heterogeneity in health behaviors among the older adult population; (2) older adults maintaining high dietary diversity coupled with regular physical activity exhibited optimal cognitive function, while physical inactivity was significantly associated with approximately 40–50% elevated cognitive impairment risk; (3) the protective effect of physical activity on cognitive function demonstrated independence—even among populations with high dietary diversity, lack of exercise significantly increased cognitive impairment risk; and (4) these associations remained robust across multiple sensitivity analyses. These findings provide important evidence for developing integrated cognitive health promotion strategies for older adults.

The findings regarding the association between dietary diversity and cognitive function in this study are generally consistent with prior literature. The Japanese NILS-LSA study, following 1,526 older adults over 12 years, found that the high dietary diversity group demonstrated significantly slower cognitive decline compared with the low diversity group (23). The French Three-City Study, following 8,085 older adults over 9 years, showed that each 1-point increase in dietary diversity was associated with an 11% reduction in dementia risk. Studies utilizing CLHLS data from our research group have reported similar findings (24). The present study, through trajectory analysis methodology, further reveals the importance of sustained maintenance of high dietary diversity (rather than single time-point measurement), providing stronger evidence supporting causal inference.

Regarding the relationship between physical activity and cognitive function, the evidence is more robust and consistent. A meta-analysis incorporating 16 prospective studies (totaling 163,797 participants) demonstrated that high-level physical activity was significantly associated with a 28% reduction in all-cause dementia risk (OR = 0.79, 95% CI: 0.69–0.88)(25). A Mendelian randomization study from the UK Biobank provided genetic evidence for a causal association between physical activity and dementia (26). A Swedish study analyzing 44 years of follow-up data demonstrated a significant inverse association between midlife physical activity levels and late-life dementia risk (27). The innovation of the present study lies in combining longitudinal trajectories of both physical activity and dietary diversity in the analysis, revealing the independence and complexity of their joint effects.

It is noteworthy that this study found the cognitive impairment risk in the “High DDS and Low PA” group (*RR* = 1.48) was even slightly higher than that in the “Low DDS and High PA” group (*RR* = 1.33, not significant), although the latter did not reach statistical significance. This finding suggests that the protective effect of physical activity on cognitive function may be more critical and direct than that of dietary diversity. Previous studies pre-dominantly focused on single behavioral factors and were unable to evaluate the relative importance of different behaviors (28). Physical activity may exert more direct and critical protective effects on cognitive function, potentially related to its multi-level biological mechanisms. Exercise directly increases cerebral blood flow and tissue oxygenation; both animal experiments and human studies have confirmed that exercise improves cerebrovascular function, increases capillary density, and promotes angiogenesis (29, 30). Functional magnetic resonance imaging studies have demonstrated that regular exercise increases blood perfusion in the hippocampus and pre-frontal cortex, regions critical for memory and executive function (31). Exercise also stimulates secretion of multiple neurotrophic factors, including brain-derived neurotrophic factor (BDNF), insulin-like growth factor-1 (IGF-1), and vascular endothelial growth factor (VEGF), promoting hippocampal neuronal survival, neurogenesis, and cerebrovascular remodeling (32–35). Exercise possesses potent anti-inflammatory and antioxidant properties, reducing circulating inflammatory marker levels and attenuating chronic low-grade inflammation in the brain (36, 37). Additionally, exercise exerts neuroprotective effects through improving insulin sensitivity and glucose metabolism, which is particularly important for delaying the progression of neurodegenerative diseases such as Alzheimer's disease (38, 39). Animal studies have further demonstrated that exercise enhances brain glymphatic system function, facilitating clearance of metabolic waste products including β-amyloid (40, 41).

In contrast, the effects of dietary diversity on cognitive function may be more indirect and long-term. A diversified diet exerts its effects through providing abundant neuroprotective components including antioxidant nutrients, B vitamins, and omega-3 polyunsaturated fatty acids (42, 43); however, absorption, metabolism, and delivery of these nutrients to brain tissue requires considerable time. The benefits of dietary diversity may also be partially mediated through improving gut microbiome composition and regulating “gut-brain axis” function (44, 45). This study found that even among populations with high dietary diversity, physical inactivity still significantly increased cognitive impairment risk, suggesting that the direct neuroprotective effects of exercise cannot be fully substituted by favorable dietary habits.

The findings of this study have important implications for developing cognitive health promotion strategies for older adults. This study found that more than one-third (34.1%) of older adults simultaneously exhibited dual unfavorable behavioral patterns of low dietary diversity and lack of regular exercise; this high-risk group should be prioritized for intervention. Targeted integrated lifestyle interventions may be more cost-effective than single-behavior interventions (46). The findings emphasize the central importance of physical activity promotion. Given that the cognitive impairment risk in the “High DDS and Low PA” group was even higher than that in the “Low DDS and High PA” group, it is recommended that physical activity interventions be prioritized when resources are limited.

This study is based on a nationally representative large-sample prospective cohort with a follow-up duration of 10 years, providing clear temporal relationships. It is the first to employ LCGA methodology to simultaneously identify longitudinal trajectories of diet and physical activity, overcoming limitations of traditional single time-point exposure assessments, and validated result robustness through multiple sensitivity analyses. Compared with traditional approaches based on single-time-point measurements or composite healthy lifestyle scores, the trajectory-based approach used in this study is able to identify the dynamic patterns of lifestyle behaviors over time. This method not only reveals distinct developmental trajectories of dietary diversity and physical activity but also evaluates the long-term impact of their joint changes on cognitive health, thereby providing new evidence for understanding the dynamic evolution of lifestyle behaviors. The study employed education-stratified cognitive impairment diagnostic criteria, reducing confounding attributable to educational attainment. This study also has certain limitations. Measurements of dietary diversity and physical activity were relatively simplified; DDS was based only on consumption frequency of nine food categories, and PA employed only a single-item assessment, resulting in potential measurement error and information loss. Future studies may utilize more detailed dietary frequency questionnaires and objective physical activity measurement tools (such as accelerometers) for validation. The entropy value (0.53) for DDS trajectories was below the ideal criterion (0.7), suggesting some uncertainty in classification; however, average posterior probabilities for each class exceeded 0.85, indicating acceptable results. The generalizability of this study's findings warrants validation in other cultural and ethnic populations.

In summary, this prospective cohort study demonstrates that sustained physical activity exerts independent and potentially more critical protective effects on cognitive function in older adults. Even among older adults maintaining high dietary diversity, lack of exercise significantly increases cognitive impairment risk. These findings underscore the importance of prioritizing physical activity promotion in cognitive health interventions for older adult populations, while also supporting implementation of integrated lifestyle intervention strategies that simultaneously improve dietary and exercise behaviors.

## 5 Conclusions

Based on 10-year prospective cohort data from the Chinese Longitudinal Healthy Longevity Survey, this study employed latent class growth analysis to identify joint longitudinal trajectory patterns of dietary diversity and physical activity, systematically evaluating associations between different trajectory groups and cognitive function in older adults. The findings demonstrate that older adults who maintained high dietary diversity coupled with regular physical activity exhibited optimal cognitive function, while physical inactivity emerged as an independent risk factor for cognitive decline, the adverse effects of which could not be fully offset by favorable dietary habits. These findings support integrated cognitive health management strategies for older adults that combine dietary intervention with physical activity promotion, and suggest that physical activity interventions should be prioritized when resources are limited. Future high-quality randomized controlled trials are needed to further validate the protective effects of integrated lifestyle interventions on cognitive function in older adults and to determine optimal intervention modalities.

## Data Availability

Publicly available datasets were analyzed in this study. This data can be found here: https://opendata.pku.edu.cn.
