# Supplementary material for: Association of joint trajectories of dietary diversity and physical activity with cognitive function in older adults: a prospective cohort study
Source: Front Public Health. 2026 Apr 16;14:1800751. doi: 10.3389/fpubh.2026.1800751 (PMC13128396; doi:10.3389/fpubh.2026.1800751)
Supplement: Supplementary file 1 [file Data_Sheet_1.docx]

Supplementary Material

Association of joint trajectories of dietary diversity and physical activity with cognitive function in older adults: a prospective cohort study

# Supplementary Data

**Table S1. LCGA fitting information of the dietary diversity score trajectories of older people**

| **Model** | **Log likelihood** | **AIC** | **BIC** | **aBIC** | **Entropy** |
| --- | --- | --- | --- | --- | --- |
| **1** | -11061.67 | 22129.33 | 22145.86 | 22139.62 | 1 |
| **2** | -10816.59 | 21645.19 | 21678.25 | 21665.77 | 0.530 |
| **3** | -10794.93 | 21607.86 | 21657.45 | 21638.74 | 0.507 |
| **4** | -10776.39 | 21576.79 | 21642.91 | 21617.96 | 0.469 |
| **5** | -10773.67 | 21577.34 | 21659.99 | 21628.80 | 0.428 |

**Table S2. LCGA fitting information of the physical activity trajectories of older people**

| **Model** | **Log likelihood** | **AIC** | **BIC** | **aBIC** | **Entropy** |
| --- | --- | --- | --- | --- | --- |
| **1** | -3862.75 | 7731.50 | 7748.03 | 7741.80 | 1 |
| **2** | -3655.23 | 7123.05 | 7156.11 | 7143.64 | 0.687 |
| **3** | -3546.52 | 7129.05 | 7178.64 | 7159.93 | 0.400 |
| **4** | -3413.53 | 7135.05 | 7201.17 | 7176.22 | 0.340 |
| **5** | -3212.80 | 6455.61 | 6538.26 | 6507.07 | 0.691 |

**Table S3. Sensitivity analyses of the association between joint trajectory groups and risk of cognitive impairment**

| **Sensitivity analysis** | **Adjusted model** | **High DDS and High PA** | | **High DDS and Low PA** | | **Low DDS and Low PA** | |
| --- | --- | --- | --- | --- | --- | --- | --- |
|  |  | **RR (95% CI)** | ***P*** | **RR (95% CI)** | ***P*** | **RR (95% CI)** | ***P*** |
| Unified cutoff  (MMSE <24) | Model 1 | 1.31(1.07-1.61) | 0.009 | 1.41(1.11-1.80) | 0.005 | 1.60(1.33-1.94) | <0.001 |
|  | Model 2 | 1.07(0.88-1.31) | 0.490 | 1.09(0.86-1.37) | 0.482 | 1.12(0.92-1.36) | 0.257 |
|  | Model 3 | 1.07(0.88-1.31) | 0.491 | 1.09(0.87-1.37) | 0.464 | 1.12(0.92-1.36) | 0.269 |
| Stricter cutoff  (MMSE <18) | Model 1 | 1.76(1.26-2.48) | 0.001 | 1.58(1.04-2.39) | 0.031 | 1.97(1.42-2.72) | <0.001 |
|  | Model 2 | 1.49(1.05-2.10) | 0.024 | 1.26(0.82-1.91) | 0.290 | 1.43(1.01-2.03) | 0.046 |
|  | Model 3 | 1.53(1.08-2.17) | 0.017 | 1.27(0.84-1.95) | 0.261 | 1.48(1.04-2.11) | 0.030 |
| Excluding baseline  MMSE <24 | Model 1 | 1.62(1.16-2.26) | 0.005 | 1.66(1.11-2.49) | 0.014 | 1.57(1.13-2.18) | 0.008 |
|  | Model 2 | 1.58(1.12-2.21) | 0.010 | 1.63(1.08-2.45) | 0.019 | 1.40(0.98-1.99) | 0.067 |
|  | Model 3 | 1.59(1.13-2.24) | 0.008 | 1.66(1.10-2.50) | 0.016 | 1.42(1.05-2.03) | 0.042 |

**Table S4. Sensitivity analyses of multiple linear regression for MMSE scores across joint trajectory groups**

| **Sensitivity analysis** | **Adjusted model** | **High DDS and High PA** | | **High DDS and Low PA** | | **Low DDS and Low PA** | |
| --- | --- | --- | --- | --- | --- | --- | --- |
|  |  | **β (95% CI)** | ***P*** | **β (95% CI)** | ***P*** | **β (95% CI)** | ***P*** |
| Excluding baseline  MMSE <24 | Model 1 | -1.39(-2.22, -0.57) | 0.001 | -1.40(-2.47, -0.32) | 0.011 | -1.81(-2.60, -1.036) | <0.001 |
|  | Model 2 | -1.04(-1.86, -0.22) | 0.013 | -0.76(-1.83, 0.31) | 0.164 | -0.82(-1.65, -0.01) | 0.047 |
|  | Model 3 | -1.07(-1.89, -0.24) | 0.011 | -0.75(-1.84, 0.34) | 0.179 | -0.86(-1.69, -0.03) | 0.042 |
| Excluding baseline  MMSE ≥28 | Model 1 | -1.85(-2.96, -0.73) | 0.001 | -1.12(-2.41, 0.17) | 0.089 | -2.17(-3.19, -1.16) | <0.001 |
|  | Model 2 | -1.50(-2.58, -0.43) | 0.006 | -0.67(-1.92, 0.57) | 0.292 | -1.68(-2.71, -0.66) | 0.001 |
|  | Model 3 | -1.55(-2.64, -0.47) | 0.005 | -0.70(-1.96, 0.57) | 0.282 | -1.75(-2.81, -0.71) | 0.001 |
